# Corporate Social Responsibility at the Micro-Level as a “New Organizational Value” for Sustainability: Are Females More Aligned towards It?

**DOI:** 10.3390/ijerph18042165

**Published:** 2021-02-23

**Authors:** Naveed Ahmad, Zia Ullah, Asif Mahmood, Antonio Ariza-Montes, Alejandro Vega-Muñoz, Heesup Han, Miklas Scholz

**Affiliations:** 1 Faculty of Management Studies, University of Central Punjab, Lahore 54000, Pakistan; naveeddgk2010@gmail.com; 2 Leads Business School, Lahore Leads University, Lahore 54000, Pakistan; chairperson.ba@leads.edu.pk; 3 Department of Business Studies, Namal Institute, Mianwali 42250, Pakistan; asif.mahmood@namal.edu.pk; 4 Social Matters Research Group, Universidad Loyola Andalucía, Escritor Castilla Aguayo, 14004 Córdoba, Spain; ariza@uloyola.es; 5 Public Policy Observatory, Universidad Autónoma de Chile, 7500912 Santiago, Chile; alejandro.vega@uautonoma.cl; 6 College of Hospitality and Tourism Management, Sejong University, 98 Gunja-Dong, Gwanjin-Gu, Seoul 143-747, Korea; 7 Division of Water Resources Engineering, Department of Building and Environmental Technology, Faculty of Engineering, Lund University, P.O. Box 118, 221 00 Lund, Sweden; miklas.scholz@tvrl.lth.se; 8 Civil Engineering Research Group, School of Science, Engineering and Environment, The University of Salford, Newton Building, Salford M5 4WT, UK; 9 Department of Town Planning, Engineering Networks and Systems, South Ural State University (National Research University), 76, Lenin Prospekt, 454080 Chelyabinsk, Russia

**Keywords:** micro-level CSR, gender, pro-environmental behavior, healthcare, organizational values, positive attitude at work

## Abstract

While prior studies have largely addressed corporate social responsibility (CSR) at a macro or institutional level, its importance at the micro or individual level is to date underexplored, especially in the context of developing economies. Further, it is not clear from the studies in the extant literature how the role of females is more important in the context of environmental management as compared to males. Similarly, micro-level CSR (MCSR) is emerging as a “new organizational value”, and the organizations that acknowledge this “new organizational value” and incorporate it into their business operations are likely to achieve sustainability objectives far better as compared to their counterparts. The present study investigates the impact of MCSR on employees’ pro-environmental behavior with the moderating effect of gender in the healthcare sector of Pakistan. The data were collected from five large hospitals in the city of Lahore through a self-administered questionnaire and analyzed using structural equation modeling (SEM) in AMOS software. A total of 533 out of 800 responses were received, which were used for data analysis of the present study. The results revealed that MCSR positively influences employee’s pro-environmental behavior, and gender moderates this relationship but the moderating effect of females is stronger as compared to males. The findings of the present study would help policymakers understand the importance of MCSR as a “new organizational value” to influence employees’ pro-environmental behavior with a special focus to promote the proactive role of females at workplaces.

## 1. Introduction

Pakistan accounts for less than one percent of the World’s greenhouse gas emission, but still, its population of more than 200 million is vulnerable to the changing climatic conditions [1]. The country has been experiencing extreme temperatures, droughts and floods, during the last two decades, and it is a continuous threat to the ranks and files of the country. According to a recent report of the Global Climate Risk Index [1], Pakistan is among the top ten countries most vulnerable to climate change. It is also expected that the current climatic conditions in the country are likely to continue in the future if serious measures are not taken at every level.

The phenomenon of corporate social responsibility (CSR) is perhaps as old as the concept of business itself. Different examples from businesses in the context of CSR can be found in the history of the ancient world [2]. As a result of the massive industrialization in Europe and other regions of the world, large enterprises came into existence and the debate on the social responsibility of the businesses began [3]. In this regard, the first book on CSR titled “*Social responsibility of the businessman*” was written in 1953 by Bowen and Johnson [4], and since then, there had been different debates on the topic of CSR in extant literature. The notion of CSR attracted policymakers throughout the globe, and several policy documents related to CSR began to emerge from several agencies. One of the references to define CSR in the literature is provided by the World Business Council for Sustainable Development (WBCSD), describing it as “CSR is the sustainable entrepreneurial orientation to achieve the economic objectives along with improving the quality of life of workers and their families and society at large” [5]. Carroll [6] proposed a four-dimensional CSR definition, consisting of economic responsibility, legal responsibility, ethical responsibility, and philanthropic responsibility.

Whereas in developed countries, the phenomenon of corporate social responsibility (CSR) has emerged as a key strategic enabler to preserve nature [7,8], the situation remains quite disappointing in developing countries. The state of CSR affairs is very poor in most developing countries, and a lot of inconsistencies in this connection have been reported by contemporary CSR researchers [9,10]. Further, most of the previous studies in the domain of CSR in developing countries context have largely addressed the relationship of CSR with a philanthropic mindset [11,12,13] but its important relationship to improve the natural environment is still under-explored. Likewise, the prior studies investigated the phenomenon of CSR at a macro or organizational level but its contribution at the individual (employee) or micro-level is something that has not received the due attention of modern researchers. Micro-level CSR (MCSR) may be very important to mitigate the environmental footprint of an organization [14]. The individuals such as employees are key players for every organization to achieve its business objectives, and ignoring their strategic role to maintain a sustainable environment is unwise [15].

Employees spend a significant amount of their time at workplaces on a daily basis, so if they are encouraged to be engaged in such activities that can limit the level of environmental dilapidation, then it can be hoped that a better and sustainable future can be achieved. Such environmental-related activities may include using less electricity, printing double sides of a paper or not taking the prints which are unnecessary, using stairs instead of the electronic escalator and like [16]. Whereas, in the context of the healthcare sector, these activities may include employees not using disposable gloves unnecessarily, not wasting water, using durables instead of disposables, using less packaging and alike [17]. Further, usage of locally produced items should be encouraged by healthcare staff because the items produced in remote areas will contribute to increasing pollution levels in the forms of logistics movement (for example, ships, aircraft, trucks etc.) These activities from employees can be expected if their behavior towards nature is improved and they realize that their individual contribution is vital to preserving nature [18]. Further, eco-feminist literature further confirms the gender differences in human and environmental relations, indicating that females are more inclined to preserve the environment [19,20]. Common sense suggests that females focus on the role of guardian and are interested in learning and nurturing social behaviors such as helping and caring for others [21,22]. Likewise, the present study contends that females are likely to be more inclined to preserve nature as compared to males.

MCSR is one such strategy that encourages employees at workplaces to be involved in such behaviors that can contribute towards a sustainable future. This study defines MCSR in line with the definition of Rupp and Mallory [23], who describe MCSR as “*MCSR is the study of CSR effects on individual levels (in any stakeholder group) to achieve sustainability*”. There have been different studies in the extant literature acknowledging MCSR as a “new organizational value” to mitigate the environmental footprint of enterprises [14,24,25] and to shape the employees’ pro-environmental behavior [26,27]. This study defines employees’ pro-environmental behavior in line with the definition of Stern [28] who defines it as “*employees’ pro-environmental behavior is the behavior of all individuals at workplace to improve environmental sustainability*”. In order to bridge the gaps in the contemporary CSR literature stated above, the present study aims to investigate the relationship between MCSR and employees’ pro-environmental behavior with the moderating effect of gender.

The proposed model of the study is tested in the healthcare sector of Pakistan, which is chosen purposefully due to three reasons. First, the healthcare sector is one of those sectors where the contribution of female workers is obvious. The majority of nurses are female and almost half of the clinicians and surgeons in Pakistan are female. Hence, healthcare is the industry with high female representation inside the patriarchal culture of Pakistan. Second, Pakistan is a patriarchal society where male dominance is evident in major decision makings. It is worth mentioning here that the female constitutes more than 48% of the total population of Pakistan and their contribution in the labor force is more than 22% [29]. However, unfortunately, their contribution to society largely remains passive due to multiple factors [30]. Hence, it will be unwise to hope for a better society and environment without the active participation of females in Pakistan. Third, the present state of CSR affairs in the healthcare sector of Pakistan is mostly philanthropic-oriented. The majority of healthcare institutes invest their CSR-related funds for charity and donations, such as treating the poor free of cost and providing free medicines and food to the poor. But, addressing the widespread issue of climate change through CSR is not their priority.

The contributions of the present study to the recent literature are many, for example, the present study enriches the existing CSR literature from the perspective of MCSR, whereas the majority of previous studies have considered this under the domain of macro or institutional level [11,12,13]. In the same vein, the present study adds significantly to existing CSR literature by proposing the relationship of MCSR to shape the pro-environmental behavior of employees at the workplace. Further, the present study enriches the recent CSR literature in the context of the service sector, whereas most of the past studies have primarily addressed the concept of CSR in the context of the manufacturing sector [31,32]. Although there have been some studies in the service sector like the banking sector [33,34], hospitality industry [35], food sector [36] but the healthcare sector in this regard remained under-explored. Lastly, the present study is the pioneer one that introduces the intervention of gender as a potential moderator in the relationship of MCSR and employees’ pro-environmental behavior.

## 2. Literature and Hypotheses

### 2.1. MCSR and Employees’ Pro-Environmental Behavior

Organizations are supposed to perform their business operations with the lowest level of negative externalities. In the same way, the organizations are expected to take care of the environment through the adoption of environment-friendly processes and the training and motivation of their workforce. The organizations undertake various mechanisms, including CSR activities, to combat environmental challenges. There are many reasons why organizations are engaged in CSR activities, for example, expectations from different stakeholders [37] and the level of competition [38]. Organizations can enrich their environmental performance when they align their CSR programs with environmental efficiency objectives, for example, low energy consumption and waste management systems to mitigate the negative impact on the environment [39]. The stakeholders, such as customers, employees, competitors and government, force the organizations to engage in CSR-related activities in order to address the environment-related issues in real senses [40].

There is an agreement among contemporary researchers that CSR is one of the most promising management strategies that produce multiple results for the organizations [41,42]. CSR is a complex and contextual phenomenon that is perceived differently in different parts of the world, and perhaps this is the reason that, to date, there is no unanimous definition of CSR that is universally accepted. In this regard, the authors are in line with the most cited CSR definition by Carroll [6] who states that “CSR comprises four sets of responsibilities for businesses, including *economic, legal, ethical and philanthropic obligations*”.

In Pakistan, as there is a dearth of literature to explain the management of environmental issues by exercising CSR activities in the domain of the environment. The prior literature in the CSR domain is mostly concerned with the impact of CSR activities on the economic performance of an organization [43,44]. This is quite recent in the literature that researchers have shown an increasing concern towards the relationship between CSR and environment management [45,46]. Other studies have also demonstrated that CSR and organizational environmental performance are positively related [39,46]. The organizations can utilize their CSR initiatives to expand their social capital and pull in consumers who back green activities. By rolling out such green moves, they are likely to increase competitive advantage [47].

The hospitals significantly contribute to environmental pollution and account for significant environmental pollution. The wastes excreted to the environment by hospitals are more dangerous and hazardous because these kinds of wastes are toxic. When the toxic elements become mixed with nontoxic wastes in the environment, the nontoxic wastes also turn into toxic ones [48], so the hospitals’ sensitivity towards environmental wellbeing is very crucial and should be ensured in either way [49]. Above all, the most unfortunate thing is that most hospital employees are not aware of the danger of environmental pollution caused by the hospital employees. The hospitals in Pakistan are overloaded and healthcare facilities in proportion to patients are terribly low. The huge influx of patients to the hospitals causes more hospital wastes, and almost the nonexistence of scientific management of the hospital wastes worsens the environmental conditions even more [50].

It is believed that CSR at a narrower or micro-level can be effective in dealing with environmental issues [51]. The healthcare sector being labor-intensive can make their employees behave in ways that cast a considerable impact on overall environmental improvement. Focusing on MCSR may produce a comparatively more pertinent outcome because each individual becomes responsible for his/her environment-related activities [27]. When CSR activities are communicated at a micro-level in an organization, it encourages the employees to develop positive behaviors on their part in order to preserve the environment. Hence, due to MCSR activities, employees are in a better state to recognize their important role in environmental management [52]. By the same token, when an organization announces CSR as one of its organizational value, and incorporates it into their core business objectives, this makes sense for the employees that their organization is showing serious concern for the environment and they need to support their organization in this cause. Thus the need to examine CSR at a micro-level in relation to environmental conditions arises. Hence the following hypothesis is framed:

**Hypothesis** **1** **(H1).** *Micro-level CSR activities promote employees at the workplace to be engaged in pro-environmental behavior.*

### 2.2. Gender as a Moderator

The present study uses the lenses of social role theory [53] and gender role theory [54] to support the role of gender as a moderator between the relationship of MCSR and employee pro-environmental behavior. Social role theory suggests that day to day roles or activities are divided into different socially defined categories in which each role has a set of expectations that an individual has to perform. Gender role theory suggests that males and females are inclined to perform different roles that are in line with social structures and are expected to be judged against a set of divergent expectations for how to behave. In the same way, the present study argues that both genders have different roles and perform differently in a social setting. Generally, females have been more caring as compared to males.

Gender role theory [54] envisages that individuals are socially identified as male and female, and tend to possess diverse attributed roles within a given social structure. Gender role is a social role encompassing a range of behaviors and attitudes that are acceptable, desirable, and appropriate for a person based on his/her biological sex. Gender roles are generally centered on the conception of masculinity and femininity. Ensuring gender equality, through retreating the several socio-economic discriminations that make females marginalized groups with meager voice and power, maybe one of the best ways of protecting the environment [55]. Commonly, females are somewhat more concerned regarding environmental issues and have stronger pro-climate and environment-friendly opinions and beliefs [56]. Females are not only sufferers from environmental degradation and climate change, but they also keep the awareness and skills that are central to finding local solutions. Therefore, the environmental programs, policies, and finance must incorporate and benefit from this knowledge and skills while supporting females in the face of contemporary unprecedented environmental experiments [57].

Some psychological and sociological researchers suggest that the reproductive and nutrient role of females establishes the outlook of caring for others and brings females high to demonstrate concerns to conserve the environment [22]. In the presence of expeditious environmental degradation, it is imperative to protect and safeguard the environment and natural resources. Females directly use natural resources, including food, fuel, water, and forest, so they have a vital role in utilizing and allocating natural resources to family, community, and the organizational level. This is the reason why females are much affected by environmental disorders [58]. Therefore, the promotion of the environment and protection of natural resources cannot be achieved without the participation of females in the activities that promote sustainability objectives of an organization.

In the healthcare industry of Pakistan, particularly in hospitals, female employees are in the majority. Some 117,000 medical doctors are female, which is 50 percent of the total doctors practicing in Pakistan. The number of dental doctors is 17,125, of which 11,039 are females. Moreover, 90% of 112,123 nurses are females. Likewise, out of a total of 62,370, the midwives and the lady health workers are female. Around 70% of the entire employees working in the hospitals of Pakistan are females. Moreover, almost 70% of the students enrolled in medical institutions are female [59].

**Figure 1:**
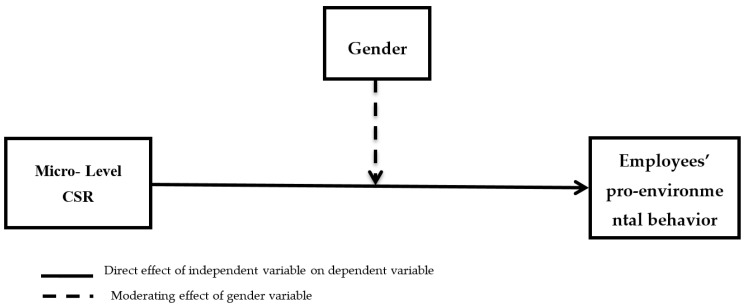
Proposed Research Model: This figure shows the path effects for H1 and H2. Where Micro-Level CSR (X) = the independent variable, Employee’s pro-environmental behavior (Y) = the dependent variable and Gender (M) = the moderating variable.

Studies on females and environment relations have shown that females have greater concern for nature and are natural resource management agents [60]. It is observed that females have a base and skill for natural resource management; they are better managers for natural resources and have sensitiveness towards environmental issues. They have high ecological consciousness, and thus, the present study proposes that gender moderates the relationship of MCSR and employees’ pro-environmental behavior (Figure 1). Hence keeping in view these theoretical assumptions and logical linkages, the authors are able to formulate the following hypothesis:

**Hypothesis** **2** **(H2).** *Gender moderates the relationship between MCSR and employees’ pro-environmental behavior such that the proposed relationship is stronger in the case of females as compared to males.*

## 3. Methods

The proposed model of the present study is tested in the healthcare sector of Pakistan. In this regard, the authors selected five hospitals in the city of Lahore including, Shaukat Khanum Memorial Cancer Hospital and Research Centre (SKMCH&RC), Pakistan Kidney and Liver Institute and Research Centre (PKLI), National Hospital and Medical Centre (NH&MC), Layton Rehmatullah Benevolent Trust Eye Hospital (LRBT) and the Indus Hospital (IH). There were two reasons for selecting these hospitals, first, these hospitals are the largest hospitals and treat a diverse set of patients for different diseases. Second, these hospitals were actively involved in CSR affairs, and each of the hospitals has a separate webpage for this purpose. Likewise, the selection of Lahore city is also logical in the context of the present study because, in recent years, Lahore has been reported as one of the most polluted city in the world [61] and requires emergency measures at every level to address this serious issue of environmental pollution.

After selecting these five hospitals, the authors contacted the spokespersons of the hospitals to get access to their staff for data collection. After repeated meetings with spokespersons, the authors got their approval to collect the data from the staff of the hospitals. In this regard, the authors signed the agreement with ethical committees of these hospitals to maintain the confidentiality of data collected from their hospitals. The wake of the second wave of COVID-19 posed some serious challenges to collect the data from hospitals, and the authors had to follow strict protocols before entering the premises of a particular hospital for the sake of data collection. The authors distributed a total of 800 questionnaires among respondents of different hospitals to collect the data. The authors also received the informed consent of respondents to maintain the data secrecy and anonymity. Finally, the authors received 533 fully filled questionnaires suitable for the data analysis, and hence the response rate of the present survey remained close to 67%.

Next, the authors dealt with common method variance (CMV) because the data for the present study were obtained from the same individual for both the independent and the dependent variables, which raises the possible existence of CMV. The authors took different steps to mitigate the potential threat of CMV. First of all, the survey questions were randomly distributed in the questionnaire in order to break any intended like or dislike preferences or sequence of the respondent for a specific variable. Second, the questionnaire items were cross-checked by the experts in the field to assure that there is no ambiguity in the questionnaire items. Third, in line with the guidelines of Fornell and Larcker [62], the authors tested the results of the single-factor analysis in SPSS in order to detect if there is any single dominant factor that is explaining more than 50% of the total variance. The results confirmed the absence of any single dominant factor because the largest variance explained by the single factor was 38.17%, which establishes that the potential issue of CMV does not exist in the present study dataset. That Table 1 presents the demographic information of the sample of the present study.

### Measures

The authors used already established scales for measuring the variables of the present study, and hence the validity and reliability of the scales is pre-established. For example, the scale of MCSR was adapted from the study of Turker [63]. This scale comprised six items (a sample item, “*This hospital participates in activities which aim to protect and improve the quality of the natural environment*”). The reliability of this scale was α= 0.76, which shows the items for this variable are consistent with each other.

Likewise, the scale of pro-environmental behavior was adapted from the study of Bissing-Olson, et al. [64], which was consisted of three items (a sample item, “I fulfill responsibilities specified in my job description in environmentally friendly ways”). The reliability of this scale was α = 0.69, which is acceptable because the scale only included three items. All items were rated on a five-point Likert scale ranging from strongly disagree to strongly agree. The authors have reported the list of items in Table 2.

## 4. Results

The data analysis phase was actuated by performing factor analysis using the SPSS software. The authors used factor analysis to confirm whether both the variable items are loading onto their respective variable and if there are some cross-loadings or weak loadings that may create problems during the phase of data analysis. The authors used principal component factor analysis (PCA) through varimax rotation to achieve this purpose. The results revealed that there is no such issue as weak loadings or cross-loading, and all the items loaded onto their respective factors with the loading of larger than 0.5 [65]. Hence, the authors established that the dataset is appropriate for analysis at a further level. Next, the authors performed confirmatory factor analysis in AMOS software in order to know if the theoretical model of the present study fits the dataset of the present study. In doing so, the authors evaluated the measurement model against the model fit indices, which are reported in Table 3 in detail. The results of different model fit indices (*χ*^2^/*df* = 4.33, RMSEA = 0.052, NFI = 0.928, CFI = 0.934, GFI = 0.929) establish that there is a good fit between the theoretical model and the database model. The authors also reported the results of validities in Table 3 for example, the convergent validity is assessed based on average variance extracted (AVE), the rule of thumb is that if the value of AVE for a particular variable is greater than 0.5, it means that the criteria for convergent validity are fulfilled. In the case of the present study variables, all values of AVEs are beyond 0.5, which means the criteria of convergent validity are well established. Likewise, the discriminant validity is established by taking the square root of AVE for each construct and comparing it with the value of correlation, for example, the square root of AVE for MCSR is 0.768, which is larger than the correlation value of 0.26 **, hence the authors establish that the variables are dissimilar with each other and the criteria of discriminant validity are well maintained. Likewise, the values of standard deviation (S.D) are less than 1 for each variable which means that there is less variability in the dataset of the present study and the data is closed to the mean values. 

Further, the authors checked the results of reliabilities (Cronbach alpha and composite reliability) through the values of (*α*) and composite reliability (CR), both values were above 0.7, which means that the internal consistency of items for both variables of the study is high, and hence the dataset qualified the criteria for reliability. Lastly, the authors also presented the results of the data normality. In this regard, the authors used the guidelines of Brown and Dacin [66], who recommended that the data is normally distributed if skewness value ranges between ±3, and Kurtosis between ±10. 

### Hypotheses Testing

The authors used the structural equation modeling (SEM) technique for hypotheses testing. In this regard, the authors used AMOS software and performed the analysis in two steps. In the first step, the results of direct effects were checked without introducing a moderating variable in the structural model. The results revealed that MCSR positively influence employees’ pro-environmental behavior (H1; *β* = 0.281 **, LLCI = 0.238, ULCI = 0.417, *p* < 0.05; *χ*^2^/*df* = 3.86, RMSEA = 0.053, NFI = 0.938, CFI = 0.944, GFI = 0.941). These results are reported in the upper path of Table 4. The results further confirmed that one unit change in MCSR would bring 0.281 ** unit of change in pro-environmental behavior (PNB). Therefore, the first hypothesis (H1) of the present study is accepted as per the support of empirical findings. In the second step, the authors introduced the variable gender as the moderator in the structural model. In order to proceed, the multi-group option in AMOS was used to create two groups of gender, like male and female, and the model was assessed for each group again. The results for both groups were significant (lower path of Table 4) but in females, the results were stronger as compared to males (*β* = 0.317 ** for females and *β* = 0.253 ** for males). These results are in line with the statement of the second hypothesis (H2) of the study and hence it is accepted that gender moderates the relationship of MCSR and PNB whereas, the group of females is more influential in this regard as compared to the group of males.

## 5. Discussion and Implications

The aim of the present research study was to investigate the impact of MCSR on employees’ pro-environmental behavior in the healthcare sector of Pakistan with the moderating effect of gender. The findings of the present study revealed that MCSR is positively related to employee’s pro-environmental behavior and gender is a potential moderator in this relationship. Sustainability has emerged as a “new normal” for modern businesses and has received attention from all sectors [67]. In this regard, modern businesses incorporate the concept of sustainability into their business operations to achieve a sustainable future world. The present study argues that employees spend a significant amount of their daily time in workplaces, and if their behavior towards nature is improved, they can contribute significantly to mitigate the intensity of environmental degradation. Further, modern organizations are required to look at MCSR as a “new organizational value” because when an organization participates in CSR activities and makes its CSR intentions clear to its employees, the employees are also encouraged to support the organization to attain CSR objectives the perspective of sustainability. The respondents of the present study confirmed that MCSR is an agent of change to shape their behavior towards the environment, and through this “new organizational value”, they were able to realize their importance at an individual level to preserve the environment. Hence, the present study establishes that MCSR has a positive relationship with employees’ pro-environmental behavior. This finding is also supported by contemporary CSR researchers [25,51,52]. The study further confirmed that the role of females is very important to achieve sustainability objectives because females are found to be more inclined towards environment management. There have been different studies in contemporary literature that also acknowledge the role of females as a guardian of nature, for example, the studies of Trelohan [55] and Galbreath [60] are some recent examples in the existing literature. The present study is also in line with the conclusions of the Davos 2021 meeting, in which global leaders have asserted the need for new policies and principles in order to address the new challenges in the world of business. The Davos agenda also recognizes the importance of collaboration from the leadership in all sectors in order to achieve sustainability objectives [68].

The present study has some important implications for theory and practice, for example, the first theoretical contribution of the present study is that it enriches the contemporary CSR literature from the perspective of microdomain of CSR, whereas the majority of previous studies considered CSR on macro-level [18,31]. Further, the present study also contributes significantly to the existing literature by proposing gender as a moderating variable between the relationship of MCSR and employees’ pro-environmental behavior in the context of Pakistan, which is a developing economy. The present study contends that there is an apparent difference between the developed and the developing nations in terms of their understanding of CSR, and it will be unwise to replicate the findings from the developed nations to a developing country like Pakistan. The authors further argue that CSR is a contextual and culture-specific concept and perhaps this is the reason that to date, there is no unanimously accepted definition of CSR. Similarly, the present study is the pioneer one to recognize MCSR as a “new organizational value” to shape the behavior of employees towards nature. Lastly, another theoretical contribution of the present study is that it considered the healthcare sector to reduce its environmental footprint in order to address the widespread issue of environmental dilapidation in the country. The study is important in the context of healthcare because the majority of the healthcare organizations in Pakistan spend their CSR fund under the domain of philanthropic orientation for example, they spend on charity and donations like activities. However, the present study argues that such a kind of spending will not help address the widespread issue of environmental degradation.

Likewise, the practical implications of the present study are also very important. First, the study suggests that policymakers from the healthcare sector of Pakistan acknowledge MCSR as a “new organizational value” to be incorporated at all organizational levels to achieve sustainability objectives. In past studies, this vital relationship of CSR at a micro level to shape the behavior of employees was largely neglected. Further, the present study brings it to the notice of policymakers to change their approach toward CSR as currently, the majority of healthcare organizations are investing their CSR funds with a philanthropic mindset. This is the time to change this mindset and rethink CSR as an agent of change to improve the current climatic conditions in the country that is a serious threat and is expected to be continued in the future as well. Similarly, the study at hand is very important from the perspective of practical implications, especially for the healthcare sector because it highlights the importance of females in improving the natural environment. In this connection, it is imperative to mention here that Pakistan is a country where male dominancy is obvious in all sectors, and the role of females largely remains passive due to male dominancy. So, this finding of the present study that females are more important to preserve nature is an eye-opener for policymakers to promote and acknowledge the importance of females if the country is hoped to have a better and sustainable future. Likewise, the policymakers should also consider the important role of males in order to achieve the sustainability objectives because the moderating role of gender is also significant as per the empirical results. Lastly, the total workforce in Pakistan is more than 75 million [69] and neglecting such a vast number to cope with the environment-related issues is unwise, so the study argues that the focus of MCSR is to encourage employees to develop volunteerism on their part to influence the environment positively.

### Limitations and Future Research Directions

The present study also has some limitations, but the authors think that these limitations open new horizons for other researchers in the same field. The first limitation of the present study is that it collected the data from only one city, and even within that city, the study included only six hospitals which raises the question of the generalizability of the present study, so future researchers should include more cities and hospitals in their datasets so that better insights can be obtained. Second, the study considered only the healthcare sector and neglected other sectors like the banking sector, insurance sector and education sector, so future researchers are encouraged to consider this limitation in their studies. Third, as human behavior is complex to understand, relying on only one factor (MCSR) to shape the behavior of employees may be risky because other variables like the role of ethical leadership, working environment, environmental training of employees are also important variables that influence the behavior of employees in the workplace. Future researchers are encouraged to introduce these variables in the present study model to understand better the influence of these variables in shaping employees’ pro-environmental behavior. Finally, the present study used cross-sectional data, which limits the ability to establish causality between the variables of the study. Hence, future researchers are encouraged to use longitudinal data in order to address the limitation of cross-sectional data.

## 6. Conclusions

To conclude, the present study is very important in the context of the current environmental vulnerability of Pakistan. This is the time to understand that efficient environmental management is only possible when every individual recognizes his or her importance and responsibility to improve the natural environment. There is a common misconception in Pakistan that CSR is the responsibility that is to be shouldered by large businesses, but now this misconception should be replaced by the correct conception that CSR belongs to everyone at all levels. Similarly, in Pakistan, the concept of CSR is primarily associated with the philanthropic domain, whereas the environmental concern in this regard is neglected that is really something to be noticed by the policymakers given the current climatic conditions of Pakistan. Likewise, policymakers are required to communicate the CSR activities of their organization with their employees as a “new organizational value” so that employees are self-motivated to observe this “new organizational value” on their part.

## Figures and Tables

**Table 1:** Demographic information of the respondents.

Demographic	Frequency	%
**Gender**		
Male	242	45.4
Female	291	54.6
**Age (Year)**		
20–25	79	14.8
26–30	193	36.2
31–40	154	28.9
Above 40	107	20.1
**Experience (Years)**		
1–3	92	17.3
4–6	211	39.6
7–10	164	30.7
Higher	66	12.4
**Healthcare professionals**		
Nurses	203	38.1
Doctors	176	33.0
**Others**	154	28.9

**Table 2:** Items with factor loadings.

Construct	Items	Factor Loading
**MCSR**	This hospital participates in activities that aim to protect and improve the quality of the natural environment	0.72
	The managerial decisions of this hospital related to the employees are usually fair	0.75
	This hospital implements special programs to minimize its negative impact on the natural environment	0.68
	The management of this hospital primarily concerns with employees’ needs and wants	0.73
	This hospital encourages its employees to participate in the voluntary activities	0.83
	This hospital contributes to campaigns and projects that promote the well-being of the society	0.87
**Pro-environmental Behavior**	I adequately complete assigned duties in environmentally-friendly ways	0.57
	I fulfill responsibilities specified in my job description in environmentally friendly ways	0.52
	I perform tasks that are expected of me in environmentally-friendly ways	0.51

**Table 3:** Correlations, validities and reliabilities.

Variable	Items		Mean	SD	MCSR	PNB		
**MCSR**	6		3.88	0.73	**0.768**	0.26 **		
**PNB**	3		4.09	0.66		**0.728**		
**Model Fit** **Indices**	**Obtained values**	**Acceptable** **values**		**AVE**	**Alpha**	**CR**	**Skewness**	**Kurtosis**
***χ*** **^2^** **/df** **RMSEA** **NFI** **CFI** **GFI**	4.17 0.069 0.910 0.933 0.931	5.00 0.08 0.90 0.90 0.90	**MCSR** **PNB**	0.59 0.53	0.76 0.69	0.78 0.69	−0.49 −0.56	0.58 0.53

**Notes:** FL: factor loading; Alpha: Cronbach’s α coefficient; CR: composite reliability; AVE: average variance extracted. Bold diagnal values = square root of AVE, ** = values are significant; PNB: pro-environmental behavior; MCSR: micro-level CSR.

**Table 4:** Hypotheses testing.

Path	Beta Value	S.E	LLCI	ULCI	Decision
	**Model 1: Standardized Direct effects**				
MCSR → PNB	0.281 **	0.037	0.238	0.417	Accepted
(*χ*^2^/*df* = 3.86, RMSEA = 0.053, NFI = 0.938, CFI = 0.944, GFI = 0.941) ***, (*R*^2^ = 0.291) * ** beta value significant, * *R*^2^ value significant					
	**Model 2: Standardized effects (moderated model)**				
MCSR → PNB (Females)	0.317 **	0.026	0.214	0.389	Accepted
(*χ*^2^/*df* = 2.35, RMSEA = 0.041, NFI = 0.963, CFI = 0.971, GFI = 0.967) ***, (*R*^2^ = 0.36) *					
MCSR → PNB (Males)	0.253 **	0.031	0.311	0.519	Accepted
(*χ*^2^/*df* = 3.11, RMSEA = 0.046, NFI = 0.958, CFI = 0.965, GFI = 0.963) ***, (*R*^2^ = 0.31) *					

CSR = corporate social responsibility, S.E **=** standard error, LLCI = lower limit confidence interval, ULCI = upper limit confidence interval. *^,^**^,^** = values are significant.

## Data Availability

The data will be made available on request from the corresponding author.
